# Liver Progenitor Cell Line HepaRG Differentiated in a Bioartificial Liver Effectively Supplies Liver Support to Rats with Acute Liver Failure

**DOI:** 10.1371/journal.pone.0038778

**Published:** 2012-06-18

**Authors:** Geert A. A. Nibourg, Robert A. F. M. Chamuleau, Tessa V. van der Hoeven, Martinus A. W. Maas, An F. C. Ruiter, Wouter H. Lamers, Ronald P. J. Oude Elferink, Thomas M. van Gulik, Ruurdtje Hoekstra

**Affiliations:** 1 Tytgat Institute for Liver and Intestinal Research, Academic Medical Center, University of Amsterdam, Amsterdam, The Netherlands; 2 Department of Surgery (Surgical Laboratory), Academic Medical Center, University of Amsterdam, Amsterdam, The Netherlands; 3 Department of Clinical Chemistry, Laboratory of Endocrinology, Academic Medical Center, University of Amsterdam, Amsterdam, The Netherlands; University of Birmingham, United Kingdom

## Abstract

A major roadblock to the application of bioartificial livers is the need for a human liver cell line that displays a high and broad level of hepatic functionality. The human bipotent liver progenitor cell line HepaRG is a promising candidate in this respect, for its potential to differentiate into hepatocytes and bile duct cells. Metabolism and synthesis of HepaRG monolayer cultures is relatively high and their drug metabolism can be enhanced upon treatment with 2% dimethyl sulfoxide (DMSO). However, their potential for bioartificial liver application has not been assessed so far. Therefore, HepaRG cells were cultured in the Academic Medical Center bioartificial liver (AMC-BAL) with and without DMSO and assessed for their hepatic functionality *in vitro* and in a rat model of acute liver failure. HepaRG-AMC-BALs cultured without DMSO eliminated ammonia and lactate, and produced apolipoprotein A-1 at rates comparable to freshly isolated hepatocytes. Cytochrome P450 3A4 transcript levels and activity were high with 88% and 37%, respectively, of the level of hepatocytes. DMSO treatment of HepaRG-AMC-BALs reduced the cell population and the abovementioned functions drastically. Therefore, solely HepaRG-AMC-BALs cultured without DMSO were tested for efficacy in rats with acute liver failure (n = 6). HepaRG-AMC-BAL treatment increased survival time of acute liver failure rats ∼50% compared to acellular-BAL treatment. Moreover, HepaRG-AMC-BAL treatment decreased the progression of hepatic encephalopathy, kidney failure, and ammonia accumulation. These results demonstrate that the HepaRG-AMC-BAL is a promising bioartificial liver for clinical application.

## Introduction

Acute liver failure (ALF) and acute-on-chronic liver failure are severe clinical syndromes with mortality rates as high as 80% [1], [2]. Clinically, the syndromes present as a severe impairment of liver function with hepatocellular necrosis, leading to hepatic encephalopathy (HE), systemic inflammation, and multi-organ failure. Despite the progress made in supportive care, liver transplantation is often the only cure, increasing the survival rates to over 80% [3], [4]. However, liver transplantation is limited by the scarcity of donor organs.In the US, about 20% of the patients with severe liver failure (MELD score >30) who are on the waiting list for liver transplantation die while waiting for a donor liver [5].

Bioartificial livers (BALs) have been developed to bridge these patients to liver transplantation or liver regeneration [6]. BALs typically comprise a bioreactor that is loaded with a biocomponent with hepatic functionality that is connected to the patient’s circulation. BAL systems, based on animal hepatocytes, are efficacious in animal models of ALF [7], [8]. However, due to xenotransplantation-related risks, there is an urgent need for BAL systems relying on human biocomponents [9].

This human biocomponent should compensate for the loss of liver function and counteract concomitant pathology, including hepatic encephalopathy (HE), inflammation, and multi-organ failure. Pathophysiologically, reduced conversion of ammonia into urea and amino acids, as well as reduced drug-metabolizing activity, lead to accumulating levels of ammonia and several other neurotoxins (*e.g.* mercaptans and endogenous benzodiazepines), which all play an important role in the progression of HE [10]. In addition, the lack of several other hepatic functions, including lactate elimination and the synthesis of blood proteins, *e.g.* apolipoprotein A-1, are associated with serious complications, such as lactic acidosis and progressive inflammation [11], [12]. Successful BAL therapy should therefore include the adequate replacement of all these functions.

The human hepatoma cell line HepaRG may be a promising candidate for BAL application. HepaRG is a bipotent liver progenitor cell line that differentiates into two distinct cell populations upon reaching confluence, optionally followed by treatment with 2% dimethyl sulfoxide (DMSO): 1) hepatocyte-like cells that self-organize into clusters; and 2) cluster-neighboring cells that express biliary markers [13], [14]. Cultured on monolayer, DMSO treatment leads to unparalleled high drug-metabolizing activity with expression of cytochrome P450 (CYP) 3A4 confined to the hepatocyte-like clusters [13]–[15].

Recently, we showed that HepaRG monolayer cultures exhibited high metabolic and synthetic properties in the absence of DMSO: the levels of galactose and ammonia elimination, apolipoprotein A-1 production, and transcription of *albumin* reached those of freshly isolated [16]. Interestingly, DMSO treatment promoted cell death and thereby reduced cell-mass more than 2-fold. In addition, DSMO treatment repressed hepatic functions originally present in cluster-neighboring regions. Nonetheless, the drug-metabolizing properties were superior in DMSO-treated cultures, with transcript levels of *CYP2B6* and *CYP3A4* reaching 61% and 69% of the levels in human liver, whereas the expression levels of these genes reached only 10% and 11%, respectively, in the −DMSO cultures. Taken together, HepaRG cells display a high and broad hepatic functionality promising for BAL application, when HepaRG cultures in the absence and presence of DMSO are being combined.

Therefore, the aim of this study was to characterize the hepatic functionality of the HepaRG cell line differentiated in the AMC-BAL, that was developed by our group, both in the presence and absence of DMSO, and to test the efficacy of this HepaRG-AMC-BAL in a rat model of ALF [17].

## Materials and Methods

### Monolayer Cell Culture and Cell Isolation

HepaRG cells, isolated from a human liver tumor [13], were cultured in Hyperflasks (Corning, New York, U.S.) in HepaRG medium without DMSO [13] for large scale expansion. Cultures were maintained at 37°C in a humidified atmosphere (95% air, 5% CO_2_). Culture medium was refreshed every 3 to 4 days. We passaged the cells every 2 weeks with a split ratio of 1∶6 (cell density ±10^5^ cells/cm^2^) using a Accutase (Innovative Cell Technologies, San Diego, U.S.), Accumax (Innovative Cell Technologies), and phosphate-buffered saline (Fresenius Kabi GmbH, Bad Homburg vor der Höhe, Germany) mix of 2∶1∶1 (v/v/v). Prior to loading the AMC-BAL, the isolated cells were centrifuged at 50× g for 5 min and washed 2× with HepaRG medium. Per AMC-BAL, a suspension of 2 mL cell pellet (approximately 750 million cells) in 9 mL HepaRG medium ( =  pre-BAL suspension) was prepared.

### AMC-BAL Culture

We used the laboratory-scale version of the third generation AMC-BAL with an internal volume of 9 mL [17], [18]. AMC-BALs were loaded with the pre-BAL suspension as described previously and from then on oxygenated with a mixture of 40% oxygen, 55% nitrogen, and 5% carbon dioxide (v/v/v) [18]. The cells were allowed to attach to the AMC-BAL’s matrix for 3 hours. Thereafter, AMC-BALs were continuously perfused with 500 mL recirculating HepaRG culture medium supplemented with 1 mM N-carbamoyl-*L*-glutamate (Sigma Aldrich, St. Louis, U.S.). This latter compound was added to increase the ammonia to urea conversion rate ∼2-fold [16]. Every 3 to 4 days, all culture medium was refreshed. We generated −DMSO BALs by culturing for 14 days on HepaRG culture medium supplemented with 1 mM N-carbamoyl-*L*-glutamate without DMSO (n = 5 to 11). The +DMSO BALs were generated by adding a 14-day culture period on HepaRG culture medium supplemented with 1 mM N-carbamoyl-*L*-glutamate and with 2% DMSO (n = 5 to 11) (Sigma Aldrich). At termination of the cultures, the BAL content was lyzed to determine DNA content [19].

### Determination of Bioactive Mass and Cell Death

To determine bioactive mass and cell death during AMC-BAL culture, we analyzed pre-BAL suspensions and total lysates of BALs for DNA content (n = 3 to 6). In addition aspartate aminotransferase (AST) and lactate dehydrogenase (LDH) activity were determined in pre-BAL suspensions and in the culture medium perfused through the bioreactor as described previously (n = 3 to 6) [19].

### Quantitative Reverse Transcription-polymerase Chain Reaction

We analyzed gene expression levels of samples of matrix containing cells (T-bags) harvested from −DMSO and +DMSO BALs as described [19], [20]. Transcript levels were normalized for 18S ribosomal RNA and expressed as a percentage of the mean of two human liver samples. These liver samples derived from two female patients, aged 40 and 41 years, with liver adenoma and no elevated liver damage, after obtaining written informed consent. The patients were not on medication and had no history of drug/alcohol abuse. The procedure was in accordance with the ethical standards of the institutional committee on human experimentation (protocol number 03/024) and the Helsinki Declaration of 1975.

### BAL Function Tests

Bioreactors were first flushed with 30 mL test medium, followed by a 24-hour period of recirculation with 100 mL of test medium (HepaRG culture medium supplemented with 1 mM N-carbamoyl-*L*-glutamate, 125 µM testosterone (Sigma Aldrich), 1.5 mM ^15^NH_4_Cl (Sigma Aldrich), 2 mM *L*-lactate (Sigma Aldrich), and 2.75 mM *D*-galactose (Sigma Aldrich)). Samples (1 mL) were taken at 0.5, 1, 2, 8 and 24 hours of recirculation and analyzed for concentrations of ammonia, urea, ^15^N-urea, 6β-hydroxytestosterone, apolipoprotein A-1, and lactate as described [16], [19], [21], [22]. Function parameter rates were determined by calculating the changes in concentration in medium per hour per BAL and per mg DNA harvested from the BALs.

### Histology

Complete transverse 8 µm sections of formaline-fixed and paraplast-embedded BALs were stained with hematoxylin (VWR, Amsterdam, The Netherlands) and azofloxine (Sigma Aldrich) as described [20].

### Rat Model of Complete Liver Ischemia (CLI)

All procedures were conducted in accordance with the institutional guidelines of the Animal Ethical Committee of the AMC (protocol number 101190). We used a rat model of CLI to induce ALF principally as described [7]. Briefly, male Wistar rats (325 to 350 g; Harlan, Horst, The Netherlands) were treated with 50 mg of the antibiotic rifaximin (Sigma Aldrich) per kg bodyweight, suspended in water, *per gavage* once a day starting from day 5 pre-CLI to standardize intestinal flora. At day 3 pre-CLI the rats were given an end-to-side portocaval shunt and the animals were allowed to recover for 3 days to prevent bleeding from the shunt anastomosis. For CLI, the hepatic artery was ligated under isoflurane anesthesia and the rats received 8 to 16 IU per 100 g bodyweight of the low molecular weight heparin dalteparine (Pfizer, New York, U.S.) intravenously (t = 0). Subsequently, the rats were allowed to wake up and move around freely. Blood glucose levels were maintained between 5 and 10 mM in all rats. In every experiment, a second rat was used as a blood donor. To this end, blood was drawn intracardially under isoflurane anesthesia and mixed with a citrate-phosphate-dextrose solution (Sanquin, Amsterdam, The Netherlands) in a 7∶1 (v/v) blood to solution proportion.

### The Extracorporeal BAL System

**Figure 1:**
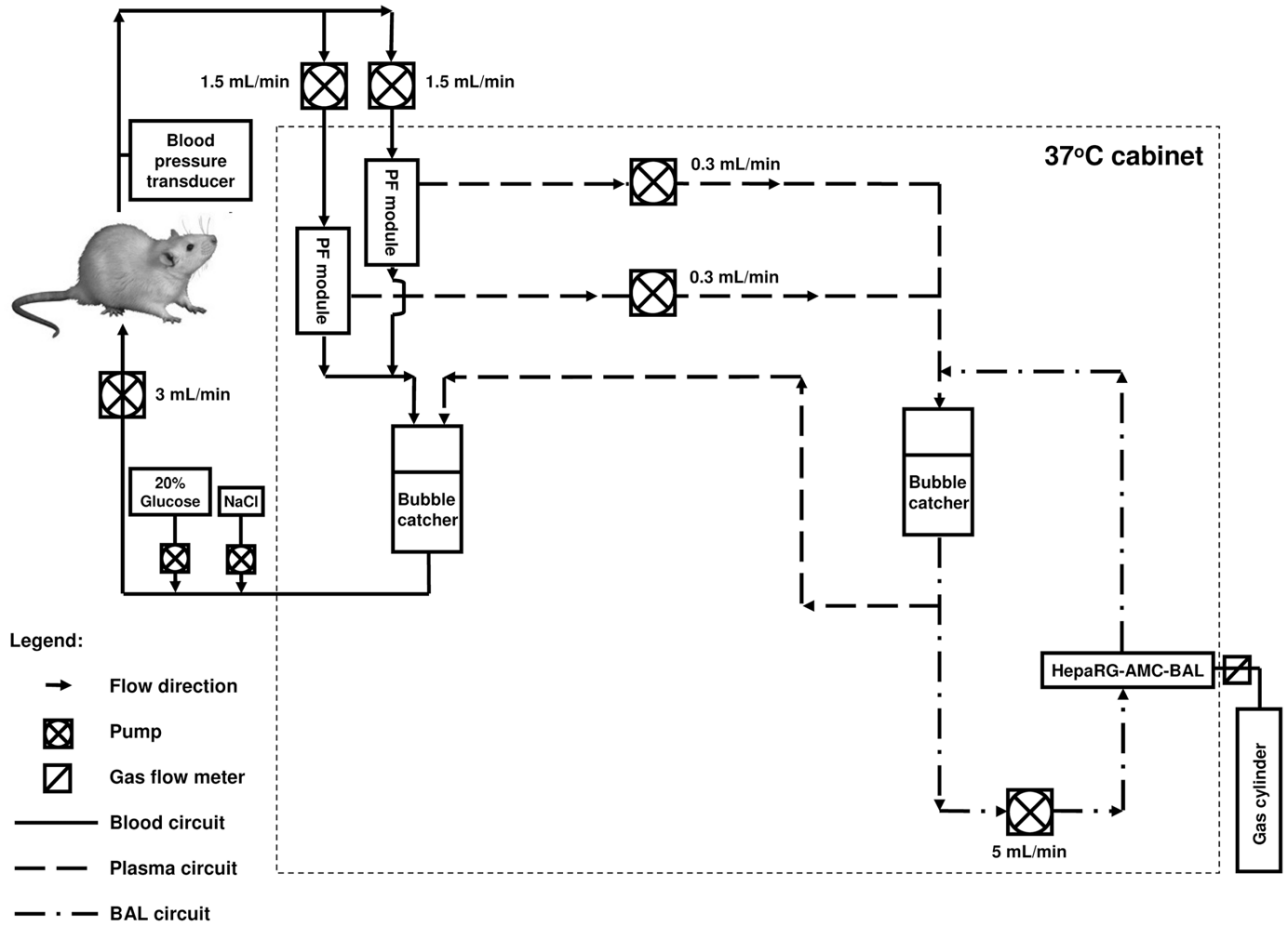
A schematic presentation of the extracorporeal bioartificial liver circuit.

We used a modified version of the extracorporeal BAL system (Fig. 1) used by Flendrig *et al*. [7]. Modifications included: 1) incorporating two, instead of one, plasmapheresis modules with an effective length of 105 mm, an internal diameter of 8 mm, 80 Plasmaphan capillaries with 0.47 µm max. pore size, and a total membrane surface of 90 cm^2^ (Membrana GmbH, Wuppertal, Germany); 2) priming of the plasma and BAL circuits of the extracorporeal BAL system with plasma expander Elohaes (Fresenius Kabi GmbH) without any supplements; 3) priming of the blood circuit with healthy rat citrate-phosphate-dextrose-blood; 4) supplementing the rat with a 20% dextrose infusion and a saline infusion to a total of 2.0 mL/h to prevent hypoglycemia and dehydration; 5) no continuous dalteparine infusion was given; and 6) placing the extracorporeal BAL system in a temperature-controlled cabinet at 37°C.

### Animal Study Design

Rats were treated with an acellular AMC-BAL (n = 9) (control group) or with a −DMSO HepaRG-AMC-BAL (n = 8) (experimental group). Exclusion criteria were: incomplete recovery from anesthesia, >6 mL intraperitoneal bleeding post mortem, hemoglobin levels <9.0 g/dL, pulmonary embolism or stroke, air embolism, and/or incomplete CLI as determined at autopsy. All these exclusion criteria were chosen to secure reproducible progression of ALF until death, without additional confounding factors, *e.g.* >50% blood loss. In the control group, the first 3 rats were excluded because of excessive (>9 mL) intraperitoneal bleeding. In the experimental group, 3 rats had to be excluded because of pulmonary embolism (thrombus formation in the tubings), air embolism (empty bubble catcher), and incomplete recovery after anesthesia. Notably, these events were unrelated to BAL treatment or the learning curve of the model. At t = 30 minutes, the rat was connected to the extracorporeal BAL system including the AMC-BAL. Blood samples of 0.1–0.5 mL were taken hourly and were replaced with healthy rat citrate-phosphate-dextrose-blood to avoid excessive blood loss. HepaRG-AMC-BALs were refreshed at t = 6 hours to prevent deterioration of BAL function due to ALF plasma toxicity. To prevent dilution by this procedure, the new BAL was filled with plasma harvested from the preceding BAL. The rat was disconnected from the extracorporeal BAL system after death and autopsy was performed. CLI was considered complete if the liver remained uncolored upon post-mortem injection of a methylene blue solution into the carotid artery, while the caval vein was clamped both inferior and superior to the liver. We did not use histopathology to validate CLI, as this method could miss small remnants of vascularized liver tissue (e.g. by anatomic variation).

### Animal Study Endpoints

The primary endpoint in this study was survival time, and secondary endpoints were the clinical HE grading score (Table 1), the blood ammonia and the plasma creatinine concentrations. Furthermore, plasma alanine aminotransferase and AST levels, hemoglobin levels, and blood glucose levels were analyzed as described [7].

**Table 1:** Clinical grading score of hepatic encephalopathy.

Score	Description
0	Normal behavior
1	Mild lethargy
2	Poor posture control; decreased motor activity; poor posture control
3	No spontaneous righting reflex; severe ataxia; diminished response to pain
4	No righting reflex on pain stimulus
5	Deep coma; no reaction on pain stimulus
6	Death

### Statistical Analysis

We compared differences between the two experimental groups using unpaired Student’s t-tests. Logrank tests were used to compare survival data. SPSS 16.0.1 (SPSS Inc., Chicago, USA) was used for statistical analysis. Prism version 4.01 (GraphPad Prism Inc, San Diego, USA) was used for graphical presentation of the data. Data are expressed as means ± standard deviations. Significance was reached if *P*<0.05.

## Results

### Proliferation, Cell Death, and Morphology of HepaRG Cells Cultured in the AMC-BAL

**Figure 2:**
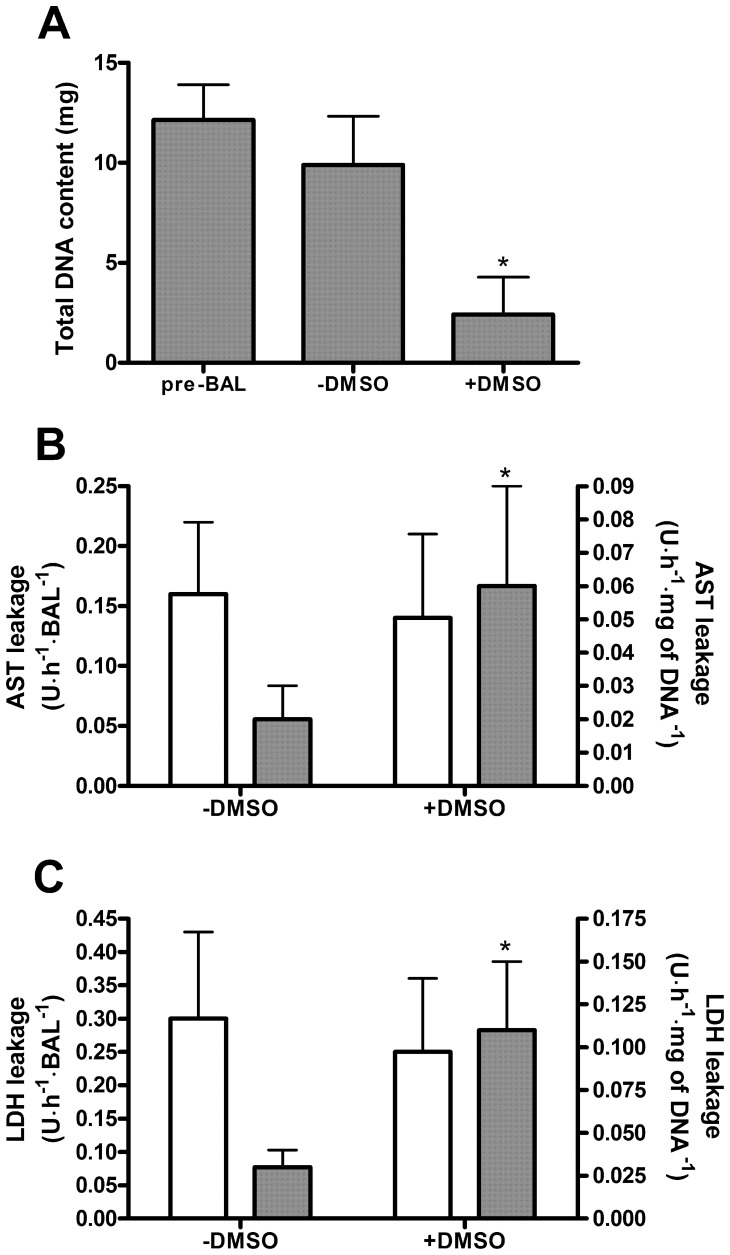
Proliferation and cell death in −DMSO and +DMSO HepaRG-AMC-BALs. Proliferation and cell death in the BALs was measured by their total DNA content (A), and leakage of AST (B) and LDH (C). AST and LDH leakage are expressed ‘per BAL’ (white bars; read along the left y-axis) and ‘per mg of DNA’ (grey bars; read along the right y-axis). Values are expressed as means ± standard deviations (n = 3 to 6). Significance: * *P*<0.05 *versus* pre-BAL suspensions (A) or −DMSO BALs (B, C).

The total DNA content at the time of loading (pre-BAL) did not significantly differ from the total DNA content of the HepaRG-AMC-BAL after 14 days of culturing without DMSO (−DMSO group) (Fig. 2A). However, in HepaRG-AMC-BALs cultured for an additional 14 days in the presence of 2% DMSO (+DMSO group), the total DNA content decreased to 25% compared to the pre-BAL and the −DMSO groups.The total AST and LDH content per BAL were 134±68 and 312±100 U, respectively. In the −DMSO BALs, AST and LDH leakage were limited, with respectively 0.16 and 0.30 U per BAL per hour, representing a leakage of 2.8% and 2.3%, respectively, of their total content per day (Fig. 2B, C). In the +DMSO BALs, AST and LDH leakage per BAL were comparable to the −DMSO BALs. At a cellular level however, this implied a 3- to 4-fold increase in cell damage in the +DMSO BALs compared to the −DMSO BALs.

**Figure 3:**
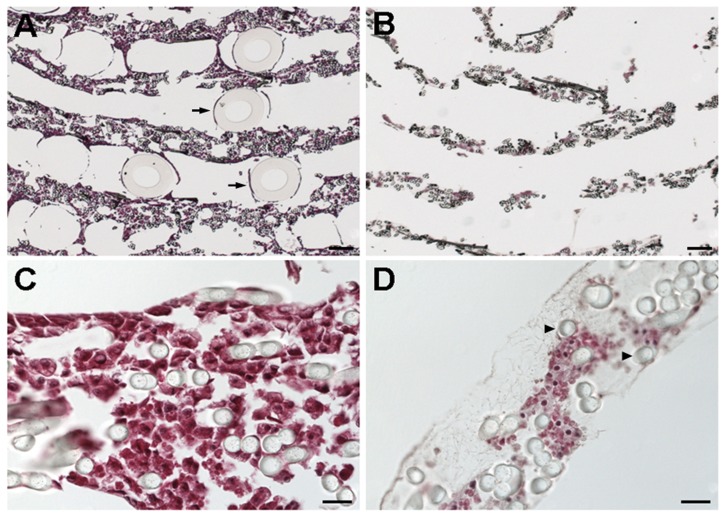
HA stainings of cross sections of −DMSO (A, C) and +DMSO HepaRG-AMC-BALs (B, D). Full transverse sections (A, B) show the spirally wound matrix layers with the gas capillaries (arrows) positioned in between. Details of the matrix (C, D) show the polyester matrix fibers (arrowheads) with HepaRG cells, and the web-shaped extracellular matrix in acellular areas in the matrix (D). Bars: 200 µm (A, B) and 20 µm (C, D).

The matrix of different cross sections of the −DMSO BALs was completely filled with cells that adhered to the polyester fibers (Fig. 3A, C). Notably, few gas capillaries were present, as they easily detach from the slides during the staining procedure. In contrast to the −DMSO BALs, the matrix of the +DMSO BALs contained many large acellular areas. A closer inspection of these acellular areas revealed the presence a web-like network of fibers typical for extracellular matrix (Fig. 3B, D).

These observations support the hypothesis that DMSO induced cell death in HepaRG-AMC-BALs. In addition, the functionality of −DMSO HepaRG-AMC-BAL remained stable until at least 28 days, excluding the possibility that prolonged culture itself (and not DMSO) could be responsible for the observed cell death (data not shown).

### In vitro Functionality of HepaRG-AMC-BALs

Transcript levels of CYP3A4 and of transcription regulators hepatic nuclear factor 4α (nuclear receptor subfamily 2, group A, member 1), and pregnane X receptor (nuclear receptor subfamily 1, group I, member 2) in the −DMSO BALs were comparable to human liver (Table 2), and only hepatic nuclear factor 4α transcript levels further increased 1.5-fold upon addition of DMSO. Interestingly, CYP3A4 transcript levels had reached 88% of human liver after 14 days culture without DMSO. Notably, CYP3A4 transcript levels reached only 10% of human liver in the −DMSO monolayer cultures, and 69% in the +DMSO cultures [16]. The CYP3A4 transcript levels increased 1.6-fold to 143% of human liver in +DMSO BALs. In contrast to CYP3A4, transcript levels of the urea cycle genes carbamoylphosphate synthetase and arginase 1 were only 29% and 16%, respectively, of human liver in −DMSO BALs, and decreased both dramatically to only 1%, upon addition of DMSO. Glutamine synthetase transcript levels were 405% of human liver in −DMSO BALs, and also decreased around two-fold upon addition of DMSO.

**Table 2:** Transcript levels of −DMSO and +DMSO HepaRG-AMC-BALs.

Gene	−DMSO	+DMSO	Fold change	*P* value
Hepatocyte nuclear factor 4α	77±31	116±8	1.5×↑	0.048
Pregnane X receptor	103±28	181±98	1.8×↑	0.066
Cytochrome P450 3A4	88±42	143±18	1.6×↑	0.076
Carbamoyl phosphate synthetase	29±7	1±0	29×↓	0.000
Arginase 1	16±11	1±1	16×↓	0.031
Glutamine synthetase	405±202	224±91	1.8×↓	0.014

Transcript levels are indicated as % of mean mRNA levels of two human liver samples and normalized for 18S ribosomal RNA. The change in transcript levels of +DMSO BALs relative to the −DMSO BALs are indicated with ↑ for upregulation and ↓ for downregulation. Abbreviations: DMSO, dimethyl sulfoxide; AMC-BAL, Academic Medical Center-bioartificial liver. Values are given as means ± standard deviations (n = 5 to 6). *P* values refer to −DMSO *versus* +DMSO BALs.

**Figure 4:**
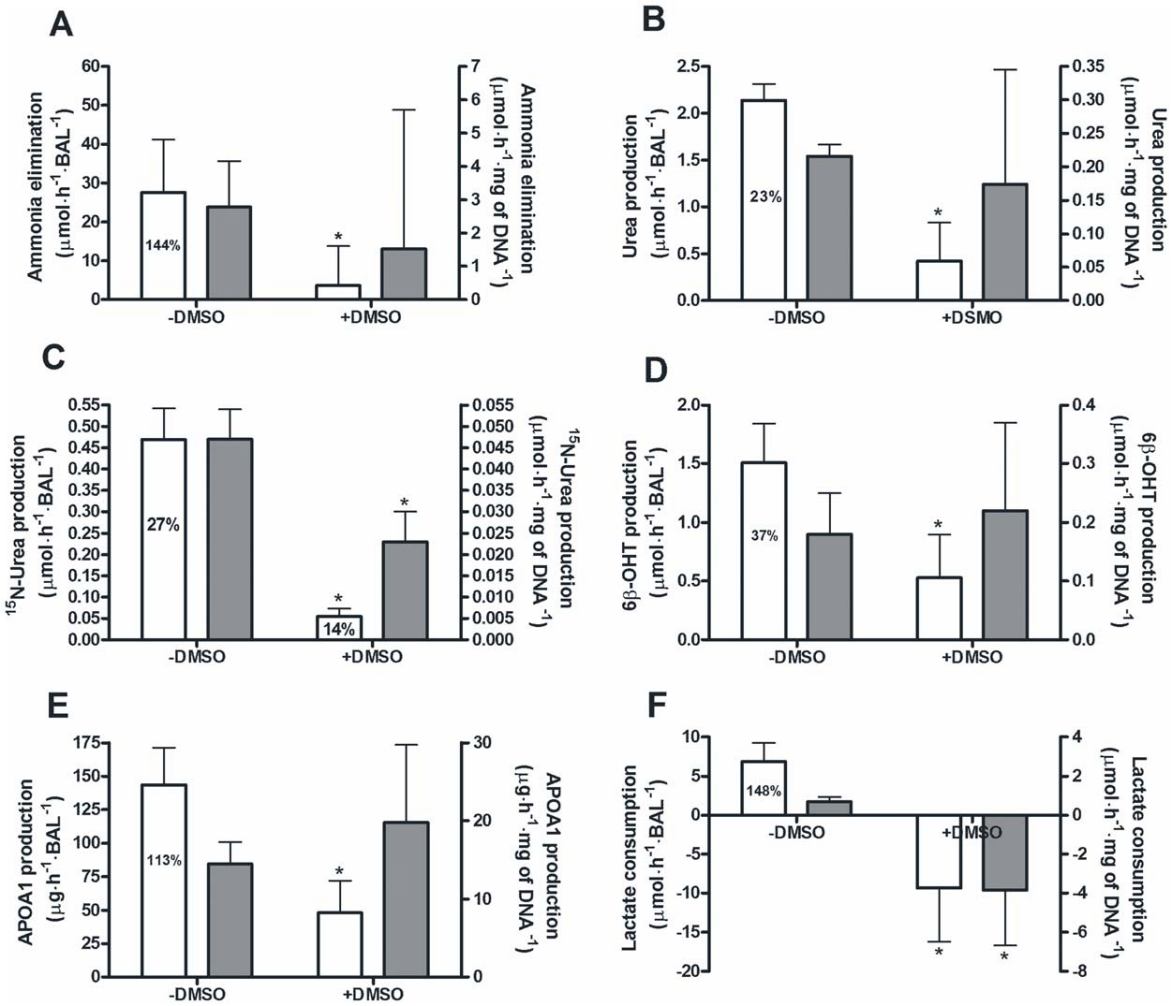
Hepatic functions of the−DMSO and +DMSO HepaRG-AMC-BALs. Ammonia elimination (A), total urea production (B), ^15^N-urea production (C), 6β-hydroxytestosterone production (D), apolipoprotein A-1 production (E), and lactate consumption (F) of −DMSO and +DMSO HepaRG-AMC-BALs expressed ‘per BAL’ (white bars; read along the left y-axis) and ‘per mg of DNA’ (grey bars; read along the right y-axis). Percentages depicted in the bars represent the percentage of this function of freshly isolated porcine hepatocytes cultured in the AMC-BAL (A, B, F) and corrected for the amount of loaded cell pellet, or of freshly isolated human hepatocytes cultured on monolayer (D, E), or the percentage mass-enriched urea of the total amount of produced urea in 24 hours (C). Values are expressed as means ± standard deviations (n = 5 to 11). Significance: * *P*<0.05 *versus* −DMSO group.

Overall hepatic functionality in −DMSO BALs was high (Fig. 4), with ammonia elimination (Fig. 4A), 6β-hydroxytestosterone production, a marker for CYP3A4 activity (Fig. 4D), apolipoprotein A-1 production (Fig. 4E), and lactate consumption rates (Fig. 4F) being between 37% and 148% of a porcine hepatocyte BAL (historical control) or freshly isolated human hepatocytes cultured in monolayer [16], [18]. Urea production was still limited with 23% of a porcine hepatocyte BAL. Moreover, analysis of the mass-enriched urea fraction revealed that 27% of the newly produced urea originated from exogenously added ammonia (Fig. 4B, C).

DMSO addition dramatically decreased the hepatic functionality per BAL for all hepatic functions measured. DMSO also reduced some hepatic functions at the cellular level, *i.e.* a 4-fold decreased conversion of ammonia into urea (Fig. 4C) and a switch from lactate consumption to lactate production (Fig. 4F). Therefore, we concluded that −DMSO HepaRG-AMC-BALs outperform +DMSO HepaRG-AMC-BALs in *in vitro* functionality and viability, and therefore only these were tested in ALF rats.

### Efficacy of the HepaRG-AMC-BAL in Rats with ALF

Two groups of rats underwent CLI: the experimental group, treated with a −DMSO HepaRG-AMC-BAL and the control group, treated with an acellular AMC-BAL. Within 45 min after induction of ALF, all animals had recovered from anesthesia. All rats developed ALF as measured by an increase in plasma alanine aminotransferase and AST levels to >15000 U/L and >20000 U/L, respectively, with no differences between both groups. Post-mortem, CLI was confirmed in all rats using the methylene blue test.

**Figure 5:**
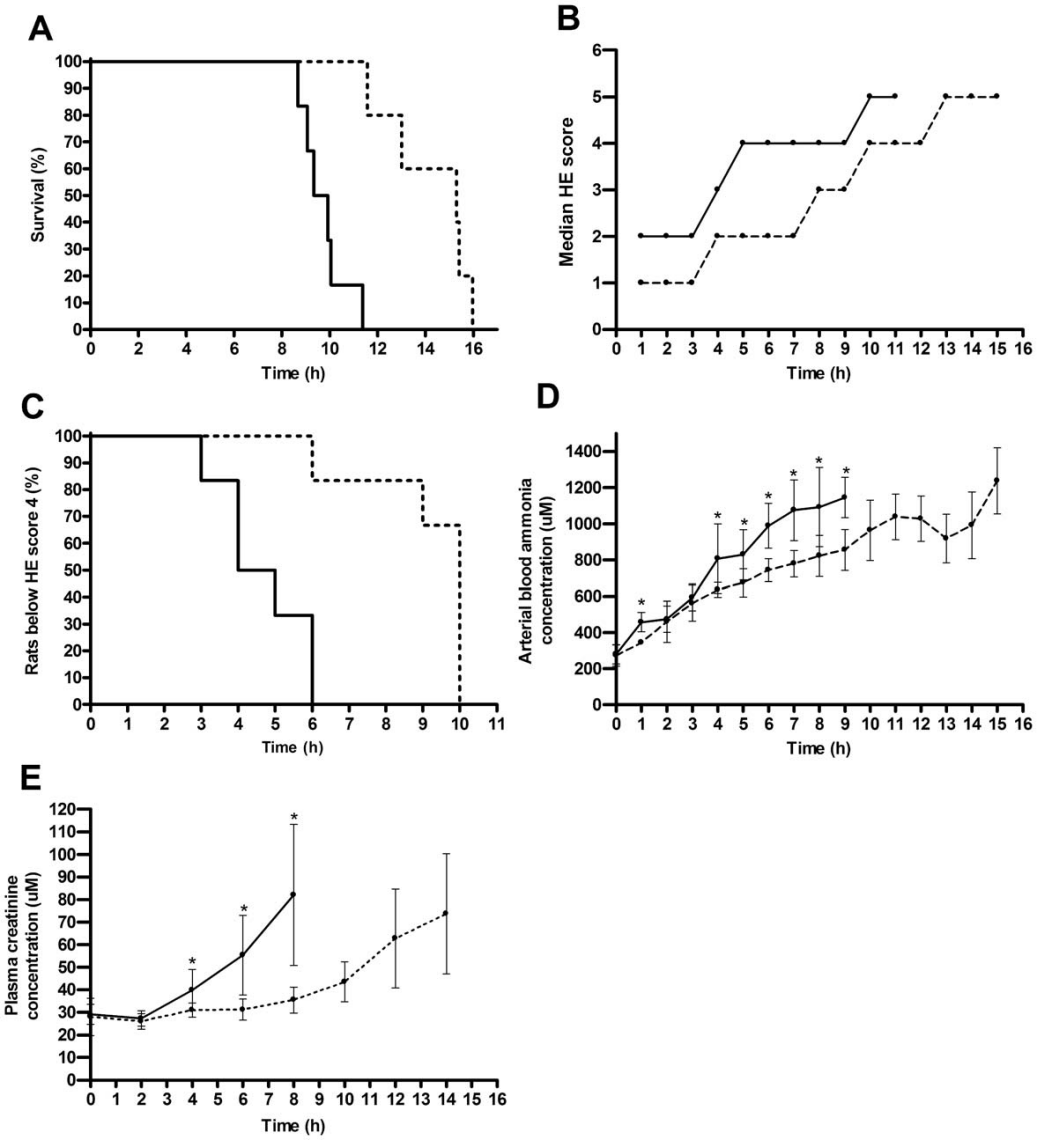
Efficacy of HepaRG-AMC-BAL treatment of rats with ALF. Efficacy was demonstrated by a significantly increased survival time (*P* = 0.001) (A), the median clinical grading score for HE (B), increased time to reach clinical HE score 4 (*P* = 0.005) (C), lower blood ammonia levels (D), and lower plasma creatinine levels (E). Continuous lines indicate the control group and dotted lines indicate the experimental group. Values are expressed as median scores (B), or means ± standard deviations (D, E) (n = 5 to 6). Significance: * *P*<0.05 *versus* control group.

The average survival time in the experimental group was 47% longer than in the control group (14.3±1.9 *versus* 9.7±1.0 hours; *P* = 0.001; n = 5 and 6, respectively) (Fig. 5A). We observed a gradual progression of HE in all rats (Fig. 5B). However, progression was significantly slower in the experimental group compared to the control group as measured by the time point at which the rats reached the easily observable HE score of 4 for the first time (4.7±1.2 *versus* 9.2±1.6 hours, respectively; *P* = 0.005; n = 6) (Fig. 5C). In all rats blood ammonia levels gradually increased to millimolar levels, but again, slower in the experimental group than in the control group, resulting in a 25% lower average blood ammonia concentration at t = 8 hours (Fig. 5D). Plasma creatinine levels were measured to assess renal dysfunction that develops secondary to ALF as part of multi-organ failure. In the experimental group, plasma creatinine levels increased slower, resulting in 57% lower concentration at t = 8 hours (Fig. 5E).

## Discussion

In this study, we demonstrated that the HepaRG-AMC-BAL displays a broad and a high level of hepatic functionality *in vitro*, as measured by the elimination of ammonia, production of urea, production of 6β-hydroxytestosterone, consumption of lactate, and synthesis of apolipoprotein A-1. In addition, we demonstrated that the HepaRG-AMC-BAL. prolongs the life of ALF rats substantially with ∼50%.

BAL culture increased hepatic differentiation of liver progenitor cell line HepaRG, particularly its detoxification functionality. CYP3A4 mRNA levels reached 88% of human liver in the −DMSO BALs, whereas this level was only 10% in −DMSO monolayer cultures and ∼70% in the presence of DMSO [16]. Hence, DMSO addition becomes superfluous for the upregulation of detoxification functionality of HepaRG cells cultured in the AMC-BAL, as opposed to HepaRG cells cultured in monolayer. This is possibly related to the increased amount of cell-cell contacts that result from the 3D culture environment of the AMC-BAL. Interestingly, a high amount of cell-cell contacts is a prerequisite for high CYP3A4 expression and activity in freshly isolated human hepatocytes [23].

In our study, DMSO addition to the culture medium had paramount negative effects on the functionality of HepaRG-AMC-BALs as a whole, probably primarily due to massive (∼75%) cell death. On the cellular level, the effects of DMSO were more ambiguous; on the one hand additional negative effects unexplained by cell death, *e.g.* lactate consumption switching into production and additional >2-fold decreased levels of CPS and ARG1 mRNA and of ^15^N-urea production, with, on the other hand increased HNF4α mRNA levels and a trend towards increased PXR and CYP 3A4 mRNA levels. The mechanism behind the differential effects of DMSO on cell functions is unknown, but might be related to its pleiotropic effect [14], [24], [25]. Proposedly, DMSO exerts its differentiating actions by inducing cessation of the cell cycle and hyperacetylation of histones. In addition, DMSO is an inducer of several phase I, II and III drug metabolizing enzymes in hepatocytes including CYP3A4 [26]. On the other hand, DMSO represses various other hepatic functions (*e.g.* expression urea cycle genes) of cluster-neighbouring cells in HepaRG monolayer cultures [16], [27], perhaps due to differential susceptibility of HepaRG cluster cells (with high CYP3A4 expression) *versus* cluster-neighbouring cells to DMSO toxicity.

*In vitro* characterization of HepaRG-AMC-BALs demonstrated that this cell line displays a level of high hepatic functionality [28]. Nonetheless, further improvements of the HepaRG-AMC-BAL can be envisaged. For example, the rate of ammonia elimination was over 50-fold higher than the rate of ^15^N-urea production, despite the addition of N-carbamoyl-*L*-glutamate to the culture medium. The metabolic fates of ammonia in the liver comprise either conversion into urea or fixation into the amino acids (glutamine and/or glutamate). As the expression of *GS* was over 400% the level of human liver, it is likely that the majority of eliminated ammonia was converted into glutamine. Glutamine in high concentrations, compromises astrocyte function in the brain and thereby contributes to the progression of HE [29]. For this reason, urea production is the preferred route for ammonia elimination. The limited conversion of ammonia into urea in the HepaRG-AMC-BAL is probably at least partly related to the low expression levels of the urea cycle enzymes *carbamoyl phosphate synthetase*, *arginase 1*, and *ornithine transcarbamoylase* [16]. Therefore, overexpressing these genes in HepaRG cells would be an interesting strategy to increase urea production.

It is difficult to speculate how an increase of ∼50% in survival time in our rat model of ALF would translate to a clinical setting of patients with ALF. However, it is important to note that, unlike in most clinical cases of ALF, the rats have no remnant liver function in our model and the HepaRG-AMC-BALs contained the equivalent of about 15%–20% of the normal total hepatocyte mass of a rat liver. This amount is too low to provide stand-alone, stable, and adequate liver support. In addition our model leads to instant and complete necrosis of the liver, eliciting massive inflammation. Notably however, our study was not devised to predict the duration of liver supporting capacity in a clinical setting, but to demonstrate the proof of principle that HepaRG-AMC-BAL therapy is efficacious in rats with ALF. To put the results of this study in perspective to BALs that use freshly isolated hepatocytes (the golden standard), the AMC-BAL using freshly isolated porcine hepatocytes as biocomponent is capable of increasing the lifespan of rats with ALF ∼2-fold using the same model of CLI [7]. However, this porcine hepatocyte BAL contained the double amount of bioactive mass compared to the HepaRG-AMC-BAL in this study, rendering comparison difficult. Notably, this difference in loading mass is caused by the ability of porcine hepatocytes to attach to the oxygen capillaries, while HepaRG cell solely attach to fibers of the matrix.

In conclusion, we have devised a human cell-based BAL using the liver progenitor cell line HepaRG as biocomponent: the HepaRG-AMC-BAL has a high and broad level of hepatic functionality and has proven efficacy in a rat model of ALF. These results encourage us to test the efficacy of the HepaRG-AMC-BAL in a clinical trial in patients with ALF or acute-on-chronic liver failure.
